# Voltammetric Determination of Penicillamine Using a Carbon Paste Electrode Modified with Multiwall Carbon Nanotubes In the Presence of Methyldopa as a Mediator

**Published:** 2017

**Authors:** Fardin Safari, Mohsen Keyvanfard, Hassan Karimi-Maleh, Khadijeh Alizad

**Affiliations:** a *Young Researcher and Elite Club, Majlesi Branch, Islamic Azad University, Isfahan, Iran. *; b *Department of Chemistry, Majlesi Branch, Islamic Azad University, Isfahan, Iran.*; c *Department of Chemical Engineering, Laboratory of Nanotechnology, Quchan University of Advanced Technology, Quchan, Iran.*

**Keywords:** Penicillamine, Multiwall carbon nanotubes, Modified electrode, Sensor, Electrocatalysis

## Abstract

A multiwall carbon nanotubes-modified carbon paste electrode (MWCNTs/MCPE) was fabricated and used to study the electrooxidation of penicillamine (PA) by electrochemical methods in the presence of methyldopa (MDOP) as a homogeneous mediator. The electrochemical oxidation of PA on the new sensor has been carefully studied. The kinetic parameters such as electron transfer coefficient, α, and catalytic reaction rate constant, K^/^_h_, were also determined using electrochemical approaches. The electrocatalytic oxidation peak current of PA showed a linear dependent on the PA concentrations and linear calibration curves were obtained in the ranges of 0.2-250.0 µM of PA concentration with square wave voltammetry (SWV) method. The detection limit (3σ) was determined as 0.1 µM. This sensor was also examined as a fast, selective, simple and precise new sensor for voltammetric determination of PA in real samples such as drug and urine.

## Introduction

Sulfhydryl based compounds (thiols, R-SH) are known to play many important roles in physiological systems (1). PA is a pharmaceutically significant thiol compound, frequently used as a therapeutic agent in several diseases such as rheumatoid artluitis and Wilson’s disease, and as an antidote in heavy metal poising (2). In Wilsonʹs disease, a rare genetic disorder of copper metabolism, PA treatment relies on its binding to accumulated copper and elimination through urine (3). Various analytical methods have been used for the determination of PA in biological and pharmaceutical samples (4-7). Among the mentioned techniques, the electrochemical methods have been very successful for determination of electroactive pharmaceutical and biological compounds (8-11). Chemically modified electrodes based on carbon nanotubes have been widely used for the electrocatalytic determination of pharmaceutical and biological compounds (12-15). The low signal using modified electrodes and high over-voltage voltammetric responses of the target analyte at unmodified electrodes are often serious problems in detection with these kinds of electrodes. A number of strategies for resolving such problems have been investigated, which are typically based on application of conductive compounds at a surface of electrode such as carbon nanotubes, nanoparticles, electroactive mediators and ionic liquids (16-20).

Electrochemical sensing and especially carbon nanotubes modified sensors have been proven as an inexpensive and simple analytical method with remarkable detection sensitivity, reproducibility, and simple preparation (22). Numerous advantages of CNTs as electrode materials have been attested for analysis of diversified chemicals of pharmaceutical, food quality, clinical and environmental interest. Carbon nanotubes modified electrode-based sensors exhibit low limit of detection, good dynamic range and fast response thanks to the signal enhancement provided by high surface area, low overvoltage, and rapid electrode kinetics (23-25).In this work, the application of MDOP as a suitable homogeneous mediator in the electrocatalysis determination of PA in an aqueous buffer solution was described initially. Following, in order to demonstrate the catalytic ability of the propose sensor in the electrooxidation of PA in real samples, the method was employed for the voltammetric determination of PA in tablet, urine samples from both patients in PA treatment and healthy subjects and on medicines.

## Experimental

*Reagents and apparatus*

All used chemicals were of analytical reagent grade purchased from Merck (Darmstadt, Germany) unless otherwise stated. Doubly distilled water was used throughout PA, methyldopa, NaOH and phosphoric acid were used from Sigma-Aldrich.

1.0 × 10^-3^ M PA solution was prepared daily by dissolving 0.015 g D-PA in water, and the solution was diluted to 100 mL with water in a 100-mL volumetric flask. The solution was kept in a refrigerator at 4 °C in dark. More dilute solutions were prepared by serial dilution with water.

Spectrally pure graphite powder (particle size < 50 µm) from Merck and multiwall carbon nanotubes (> 95%, MWCNTs, *d* × *l* = (100 – 70 nm) × (5 – 9 μm)) from Fluka were used as the substrate for the preparation of the carbon paste electrode. High viscosity paraffin (*d* = 0.88 kg L^–1^) from Merck was used as the pasting liquid for the preparation of the paste electrodes.

Britton-Robinson buffer or Universal buffer (boric acid, phosphoric acid, acetic acid plus sodium hydroxide, 0.04 M) solutions with different pH values were used.

All voltammetric measurements were performed using an Autolab PGSTAT 302N, potentiostat/galvanostat (Utrecht, The Netherlands) connected to a three-electrode cell, Metrohm (Herisau, Switzerland) Model 663 VA stand, linked with a computer (Pentium IV, 1,200 MHz) and with Autolab software. A platinum wire was used as the auxiliary electrode. MWCNTs/MCPE and Ag/AgCl/KCl_sat_ was used as the working and reference electrodes, respectively. The electrodes were characterized by scanning electron microscopy (SEM) (Seron Tech. AIS 2100). 

*Preparation of the electrode*

In the first step and for eliminating any metal oxide catalysts within the nanotubes, multiwall carbon nanotubes were refluxed in 2.0 M HNO_3_ for 18 h, and then washed twice with distilled water and dried at room temperature. 0.9 g graphite powder was dissolved in diethyl ether and hand mixed with 0.100 g CNTs with a mortar and pestle. The solvent was evaporated by stirring. A syringe was used to add paraffin to the mixture, which was mixed well for 50 min until a uniformly wetted paste was obtained. The paste was then packed into a glass tube (diameter 0.09 cm^2^). Electrical contact was made by pushing a copper wire down the glass tube into the back of the mixture. When necessary, a new surface was obtained by pushing an excess of the paste out of the tube and polishing it on a weighing paper.

*Preparation of real samples*

For the determination of PA in pharmaceuticals (capsule), seven capsules of PA labeled with 250 mg per capsule were completely grounded and homogenized. Then, 150 mg of the powder was accurately weighed and dissolved in 5 mL of water, and then the mixture was filtered on a 0.45 mm filter. The resulted solution was diluted 5 times with the buffer (pH 5.0) and used for the determination of PA contents.

Urine samples were stored in a refrigerator immediately after collection. Ten milliliters of the sample was centrifuged for 30 min at 1500 rpm. The supernatant was filtered out using a 0.45 µm filter and then 5.0 mL of the result solution mixed with 5.0 mL of the buffer (pH 5.0). The solution was transferred into the voltammetric cell to be analyzed without any further pretreatment. The standard addition method was used for the determination of PA in real samples.

**Figure 1:**
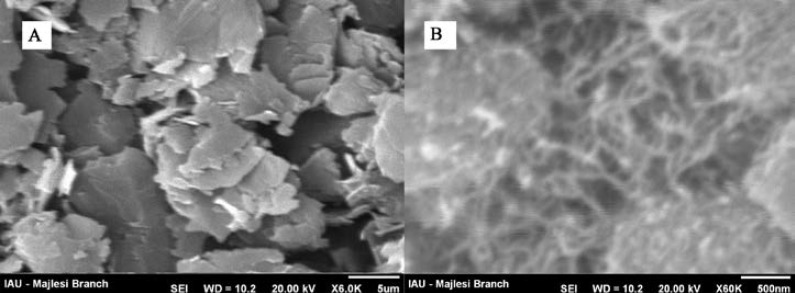
SEM images of (a) CPE and (b) MWCNTs/MCPE*.*

**Figure 2:**
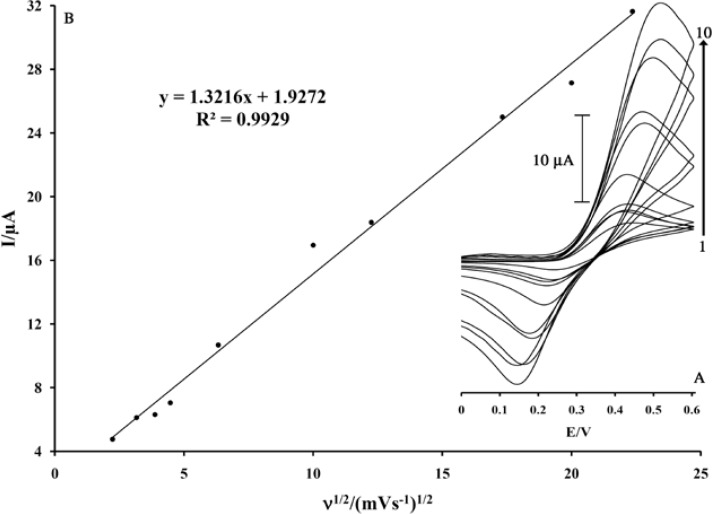
Plot of I_pa_ versus ν^1/2^ for the oxidation of 500 μM MDOP at a surface of MWCNTs/MCPE. Insert; cyclic voltammograms at various scan rates, (1) 5; (2) 10; (3) 15; (4) 20; (5) 40; (6) 100; (7) 150; (8) 300; (9) 400 and (10) 400 mV s^–1^ in 0.04 M universal buffer solution (pH 5.0).

**Figure 3:**
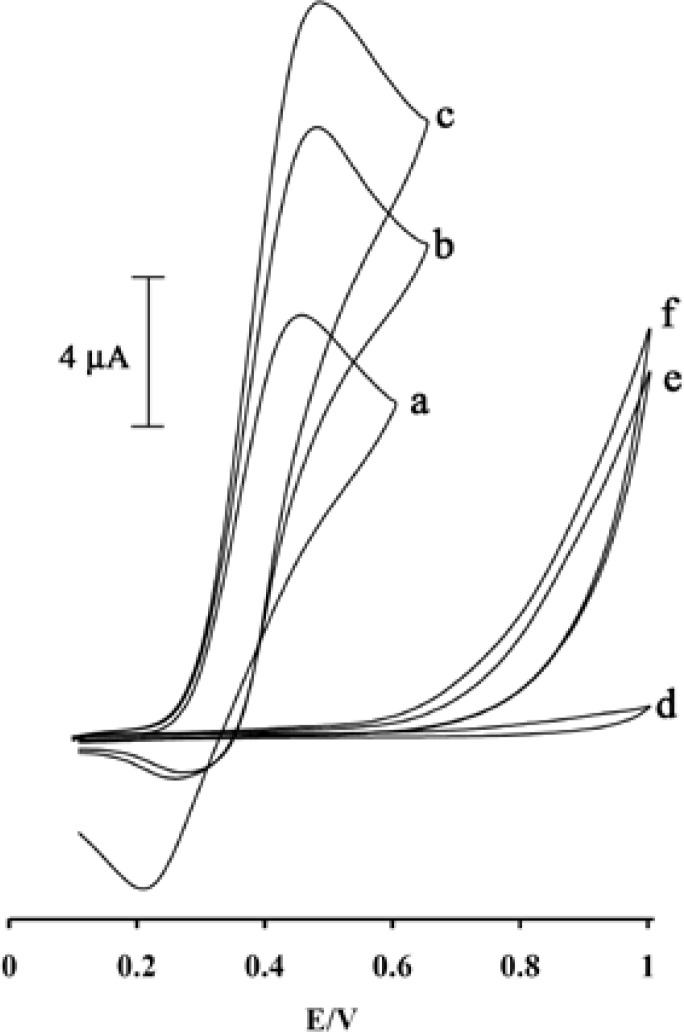
Cyclic voltammograms of (a) 500 μM MDOP at the surface of MWCNTs/MCPE in 0.04 M universal buffer solution (pH 5.0), (b) 500 μM MDOP + 100 μM PA at the surface of carbon paste electrode, (c) 500 μM MDOP +100 μM PA at the surface of MWCNTs/MCPE, (d) MWCNTs/MCPE in 0.04 M universal buffer solution, (e) 500 μM MDOP PA at the surface of carbon paste electrode, (f) 500 μM MDOP +100 μM PA at the surface of MWCNTs/MCPE; scan rate of 20 mV s^−1^.

**Figure 4:**
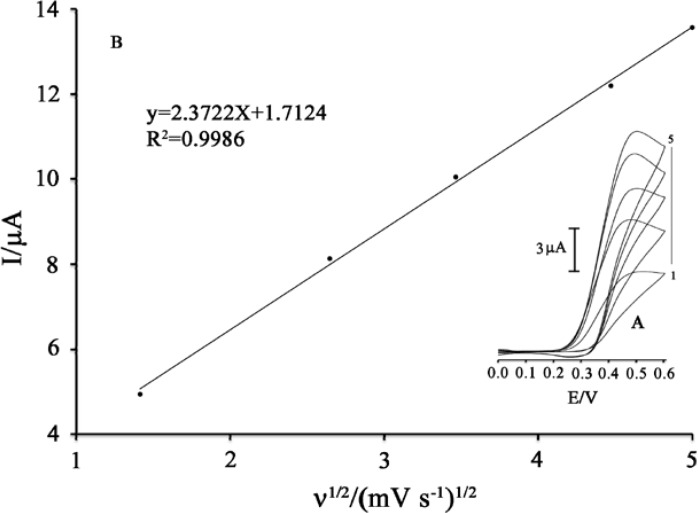
(A) Cyclic voltammograms of 100 μM PA in the presence of 500 μM MDOP at various scan rates: (a) 2, (b) 7, (c) 12; (d) 20; (e) 25 mV s^−1^ in 0.04 M universal buffer solution (pH 5.0). (B) Plot of I_pa_ versus ν^1/2^ for the oxidation of 100 μM PA in the presence 500 μM MDOP at the surface of MWCNTs/MCPE

**Figure 5:**
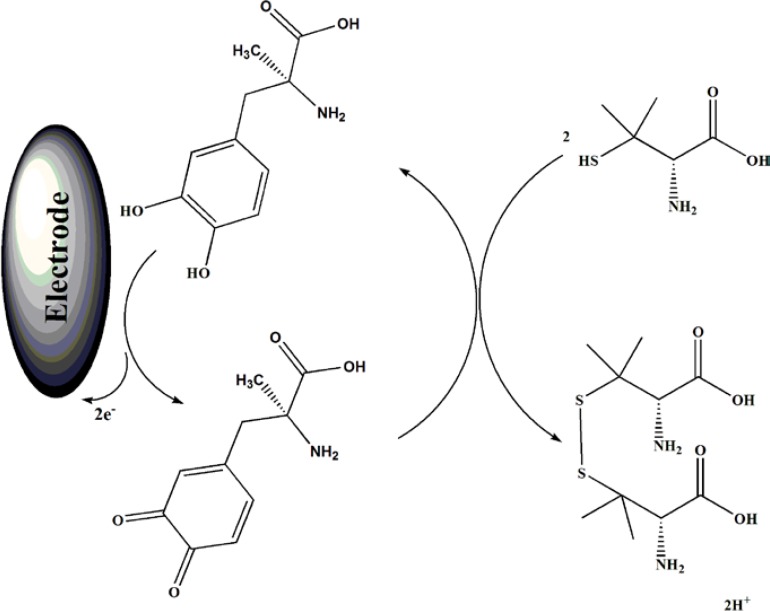
Tafel plot of 500 μM MDOP at the surface of MWCNTs/MCPE in 0.04 M universal buffer solution (pH 5.0) at a scan rate of 25 mV s^−1^ in the presence of 100 μM PA

**Figure 6:**
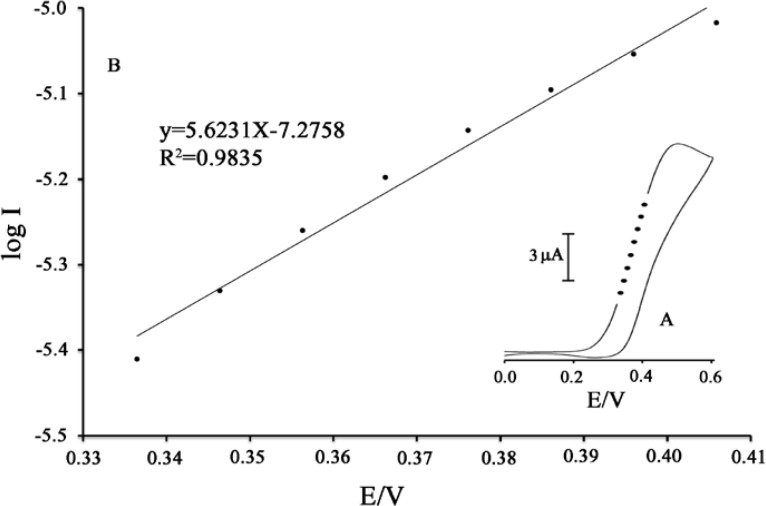
Plots of the electrocatalytic peak current as a function of PA concentration in the range of 0.2-250 μM. Inset: modified electrode SWVs in 0.04 M universal buffer solution (pH 5.0) containing different concentrations of PA

**Scheme 1:**
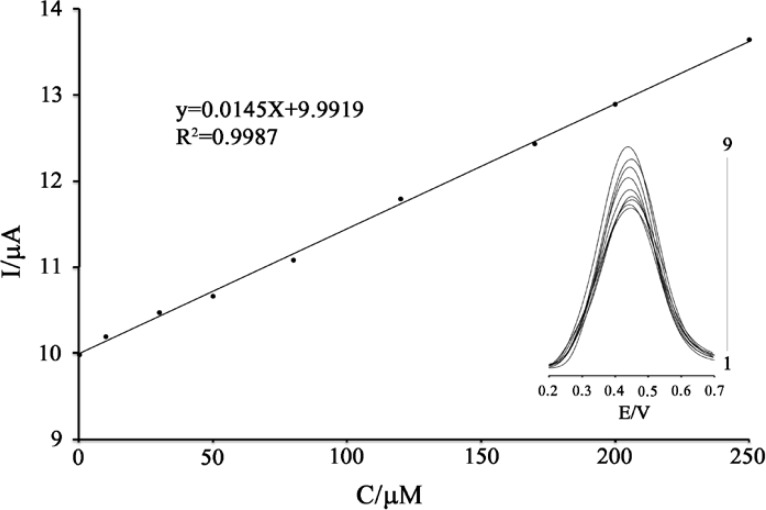
Electrocatalytic mechanism for determination of PA at the surface of MWCNTs/MCPE in the presence of the mediator

**Table 1:** Comparison of the efficiency of some electrochemical methods in the determination of PA

**Electrode**	**Modifier**	**LOD** **(µM** ^1^ **)**	**LDR** **(µ** **M** ^1^ **)**	**Ref.**
Carbon paste	P-Aminophenol	0.1	0.4-200	(41)
Carbon paste	FC-derivative	0.01	0.06-140	(42)
Carbon paste	FC-derivative	3.9	7.0-230.0	(43)
Carbon paste	Methyldopa	0.1	0.2-250	This work

**Table 2:** Interference study for the determination of 5.0 µM PA under the optimized conditions

**Species**	**Tolerante limits** **(S**_ubstance_ **/W**_PA _ **)**
Glucose , Fructose, Lactose, Sucrose, Valine, Methionine, Glycine, Lucine, Histidine, Glutamic acid, Alanine , Glycine, Phenylalanine	900
Na^+^, NO_3_^-^, Cl^-^, SO_4_^+2^, ClO_4_^-^, K^+^, Li^+^, CO_3_^2-^, F^-^, SCN^-^, Br^-^, Mg^2+^	800
Thiourea, urea, Tryptophan, Caffeine, Methanol, Ethanol	600
Starch	Saturation
Ascorbic acid^*^	500

* After addition 1.0 mmol/L ascorbate oxidase

**Table 3:** Determination of PA in tablet and urine samples (n=3).

**Sample**	**PA added** **(µM** **)**	**Expected value** **(µM** **)**	**PA founded** **(µM** **)**	**Published Method** **(µM** **)**	**t**_ex_	**t**_tab_	**F**_ex_	**F**_tab_
Tablet	−−	5.0	5.2 ± 0.4	4.8 ± 0.5	1.9	3.8	7.5	19.0
	15.0	20.0	19.6 ± 0.7	20.8 ± 1.0	---	---	---	---
	15.0	45.0	45.8 ± 0.9	45.8 ± 1.0	---	---	---	---
Urine	−−	−−	<Limit of detection	<Limit of detection	---	---	---	---
	10.0	10.0	10.6 ± 0.8	10.9 ± 0.9	2.0	3.8	8.5	19.0
	10.0	20.0	20.7 ± 0.8	21.0 ± 1.1	---	---	---	---
Urine ^a^	−−	−−	4.9 ± 0.9	5.2 ± 0.3	1.5	3.8	6.5	19.0

**F**_ex_ calculated F value; reported F value from F-test table with 95% confidence level and 2/2 degrees of freedom

**t**_ex_ calculated t; ttab(95%); reported t value from Student t-test table with 95% confidence level.

*Recommended procedure*

The MWCNTs/MCPE was polished with a white and clean paper. To prepare a blank solution for analysis, 20.0 mL of buffer solution (optimum condition; pH 5.0), was transferred into an electrochemical cell. The initial and final potentials were adjusted to –0.2 and +0.70 V, respectively. Different amounts of PA solution were added to the cell, using a micropipette, and the SWV was recorded again to get the analytical signal (I_ps_) in the presence of mediator. Calibration curves were constructed by plotting the catalytic peak current vs. the PA concentration.

## Results and Discussion

*SEM Characterization*

The morphology of the unmodified and nanostructures modified electrodes were characterized by SEM. Figure 1 shows SEM images for MWCNTs/MCPE and carbon paste electrode (CPE). As can be seen at a surface of CPE (Figure 1A), the layer of irregularly flakes of graphite powder was present and isolated with each other. After multiwall carbon nanotubes added to carbon paste, it can be seen that MWCNTs were distributed on the surface of electrode with special three-dimensional structure (Figure 1B), indicating that the MWCNTs were successfully modified on the MWCNTPE. 

*Electrochemical investigation*

As MDOP is soluble in an aqueous solution, it can be easily used as a homogeneous mediator for electrocatalytic process. The electrochemical behavior of MDOP at a surface of MWCNTs/MCPE was investigated. The cyclic voltammograms of the modified electrode in 0.04 M universal buffer (pH 5.0) are shown in Figure 2 insert. As can be seen, the cyclic voltammogram exhibits an anodic peak at the forward scan of the potential related to the oxidation of the MDOP _(Red)_ to MDOP _(Ox)_ form. In the reverse scan of the potential, a cathodic peak appears related to the reduction of MDOP _(Ox)_ form, to MDOP _(Red)_. A pair of quasi reversible peaks are observed at *E*_pa_ = 0.45 V and *E*_pc_ = 0.19 V vs. Ag/AgCl. The half-wave potential (*E*_1/2_) was 0.32 V vs. Ag/AgCl and Δ*E*_p_ (*E*_pa_ −*E*_pc_) was 0.26 V.

The electrode process was quasi reversible, with Δ*E*_p_, greater than the expected value (59/n mV) for a reversible system. The plot of the anodic peak current was linearly dependent on *ν*^1/2^ for all scan rates (Figure 2). This behavior indicates that the nature of the redox process is diffusion controlled.as we know, the electrochemical behavior of PA and methyldopa depend on pH value of the aqueous solution. So, it is very necessary for optimization of pH value to obtain the best condition in electroanalysis of PA. to this end, the electrooxidation behavior of PA in 0.04 M universal buffer solution with various pH (3.00 < pH < 6.00) at the surface of modified electrode were studied. The Result showed, the maximum electrocatalytic current was obtained at pH 5.0. Therefore, all of the electrochemical experiments were done at this pH.

Figure 3 depicts the cyclic voltammogram responses for the electrochemical oxidation of 100 µM PA at modified electrode in the presence of MDOP (curve c), carbon paste electrode (CPE) in the presence of MDOP (curve b) and MWCNTs/MCPE and CPE without MDOP (curve f and e), respectively.

Result shows, while the anodic peak potential for PA oxidation at the MWCNTs/MCPE, and CPE in the presence of mediator is ≈900, the corresponding potential at MWCNTPE and CPE without mediator are ≈497 mV. These results indicate that the peak potential for PA oxidation at the MWCNTs/MCPE, and CPE in the presence of mediator shifts by 403 mV toward negative values compared to MWCNTs/MCPE and CPE (without MDOP), respectively. However, MWCNTs/MCPE in the presence of mediator shows a much higher anodic peak current for the oxidation of analyte compared to CPE (with MDOP), indicating that the combination of CNTs and the MDOP has significantly improved the performance of the electrode toward PA oxidation. On the other hand, MWCNTs/MCPE (with mediator in the solution) in the absence of PA exhibited a well-behaved redox reaction (Figure 3, curve a) in 0.04 M universal buffer solution (pH 5.0). However, there was a drastic increase in the anodic peak current in the presence of 100 µM PA (curve b or c), which can be related to the strong electrocatalytic effect of the mediator towards this compound (see scheme 1) (26-28). 

The effect of scan rate on the electrocatalytic oxidation of PA at the MWCNTs/MCPE and in the presence of 500 µM MDOP as a mediator was investigated by cyclic voltammetry (Figure 4A). Result shows the oxidation peak potential shifted towards a more positive potential with increasing scan rate, confirming the kinetic limitation of the electrochemical reaction 

(29-32).

In addition, a plot of peak height (*I*_p_) against square root of scan rate (*ν*^1/2^) was constructed (Figure 4B) which was found to be linear, suggesting that, at sufficient over potential, the process is diffusion rather than surface controlled (33-35). In order to obtain information about the rate-determining step, Tafel plots (plots of log I *vs.* potential) were drawn (Figure 5) which were derived from points of the Tafel region of the cyclic voltammogram in Figure 5 (insert). 

The results of the polarization studies for electro-oxidation of PA at the MWCNTs/MCPE in the presence of mediator showed that for all potential sweep rates, the average Tafel slope was 5.6231 *V*^-1^ decade. The slope of the Tafel plot was equal to *n*(1 − *α*)*F/*2.3*RT*. We obtained *nα* equal to 0.67. Assuming *n* = 1, then *α* = 0.67. In addition, the value of αnα was calculated for the electro-oxidation of PA at pH 5.0 for modified electrode in the presence of mediator using the following equation:

αnα = 0:048/ (EP-E_P/2_)                    (1)

where E_P/2_ is the potential corresponding to I_P/2_. The value for αnα was found to be 0.66.

Chronoamperometry results were obtained for the various concentrations of PA solution using MWCNTs/MCPE and in the presence of 500 µM MDOP (Not shown). The plot of current (I) versus t^-1/2^ for PA solution at various concentrations using MWCNTs/MCPE and in the presence 0f 500 µM MDOP gives straight lines with different slopesFrom the slopes, a diffusion coefficient of 5.9×10^−4^ cm^2^ s^−1^ for PA using the Cottrell equation was calculated. 

Chronoamperometry can also be employed to evaluate the catalytic rate constant, *k*_h_ for the reaction between PA and MWCNTs/MCPE and in the presence 0f 500 µM MDOP according to the method of Galus (36):

*I*_C_ */I*_L_ *=γ* ^1/2^ *[π* ^1/2^ *erf (γ* ^1/2^ *) + exp(−γ)/γ* ^1/2^ *]*                      (2)

Where *I*_C_ is the catalytic current of PA at MWCNTs/MCPE and in the presence of 500 µM MDOP, *I*_L_ is the limiting current in the absence of PA and *γ=k*_h_*C*_b_*t* (*C*_b_ is the bulk concentration of PA) is the argument of the error function. In the cases where *γ* exceeds two the error function is almost equal to 1 and therefore, the above equation can be reduced to:

*I*_C_ */I*_L_ *= γ* ^1/2^ *π* ^1/2^ *=π* ^1/2^ *(k*_h_ *C*_b_ *t)* ^1/2^                     (3)

Where t is the time elapsed in seconds. The above equation can be used to calculate the rate constant of the catalytic process k_h_. Based on the slope of *I*_C_*/I*_L_ *vs.* *t*^1/2^ plot, *k*_h_ can be obtained for a given PA concentration. From the values of the slopes, an average value of *k*_h_ was found to be *k*_h_= 5.83 × 10^2^ mol^−1^ L s^−1^. The value of k_h_ explains the sharp feature of the catalytic peak observed for catalytic oxidation of PA at the surface of MWCNTs/MCPE and in the presence of 500 µM MDOP.

Double potential step chronocoloumetry, as well as other electrochemical methods, was also employed for the investigation of electrode processes at MWCNTs/MCPE and in the presence of 500 µM MDOP (Not shown). 

Forward and backward potential step chronocoloumetry on the modified electrode in a blank buffer solution showed very symmetrical chronocolougrams. These had about an equal charge consumed for both oxidation and reduction of the quinone/hydroquinone redox system in MDOP. However, in the presence of PA, the charge value associated with forward chronocoloumetry was significantly greater than what was observed for backward chronocoloumetry. This behavior is typical of what expected for electrocatalysis of chemically modified electrodes (37-40).

*Calibration plot and detection limit*

Since square wave voltammetry (SWV) has a much higher current sensitivity and better resolution than cyclic voltammetry, the SWV was used for the determination of PA (Figure 6 inset). SWVs clearly show that the plot of the peak current *vs.* PA concentration is linear for 0.2 – 250 µM of PA, with a regression equation of I_p_(µA) = (0.0145 ± 0.0025) C_PA_ + (9.9919 ± 0.9235) (r^2^ = 0.9989, n = 9), where C is µM concentration of PA, and I_p_ is the peak current (Figure 6). The detection limit (LOD) was 0.1 µM PA according to the definition of LOD = 3s_b_/m (where S_b_ is the standard deviation of the blank signal (n = 12) and m is the slope of the calibration. Comparisons of the results from the different methods are shown in Table 1.

*Stability and reproducibility*

The repeatability and stability of MWCNTs/MCPE and in the presence of 500 µM MDOP was investigated by SWV measurements of 10.0 µM PA. The relative standard deviation (*RSD%*) for seven successive assays are 1.4%. When using five different electrodes, the *RSD* for four measurements is 2.0%. When the electrode stored in our laboratory at room temperature, the modified electrode retains 97% of its initial MWCNTs/MCPE and in the presence of 500 µM MDOP has good stability and reproducibility, and could be used for PA.

*Interference study*

The influence of various substances as potential interfering compounds with the determination of PA was studied under the optimum conditions with 5.0 µM PA at pH 5.0. The potentially interfering substances were chosen from the group of substances commonly found with PA in pharmaceuticals and/or in biological fluids. Tolerance limit was defined as the maximum concentration of the interfering substance that caused an error less than ±5% for the determination of PA. The results are presented in Table 2. 

*Real sample analysis*

Electrochemical methods are powerful techniques for determination of pharmaceutical and biological compounds in real samples (44-52). In order to demonstrate the ability of the modified electrode to the determination of PA in real samples, determination of PA in pharmaceutical and in urine samples were examinedwhose results are given in Table 3. These results demonstrated the ability ofproposing sensor for voltammetric determination of PA with high selectivity and good reproducibility.

## Conclusion

This research describes the ability of the MWCNTs/MCPE in the presence of 500 µM MDOP as a suitable mediator for electro-catalytic determination of PA. The proposed sensor is highly sensitive to PA levels as low as 0.1 μM. Under the best conditions in square wave voltammetric determination, the oxidation peak current was proportional to the PA concentration in the range of 0.2–250 μM. 

The kinetic parameter of the electrocatalytic process and the diffusion coefficients of PA in an aqueous solution were determined. Finally, the proposed sensor was also examined as a selective, simple, and precise electrochemical sensor for the determination of PA in real samples such as drug and urine.
